# MBL-II-141, a chromone derivative, enhances irinotecan (CPT-11) anticancer efficiency in ABCG2-positive xenografts

**DOI:** 10.18632/oncotarget.2566

**Published:** 2015-01-20

**Authors:** Mylène Honorat, Jérôme Guitton, Charlotte Gauthier, Charlotte Bouard, Florine Lecerf-Schmidt, Basile Peres, Raphaël Terreux, Héloïse Gervot, Catherine Rioufol, Ahcène Boumendjel, Alain Puisieux, Attilio Di Pietro, Léa Payen

**Affiliations:** ^1^ Institut des Sciences Pharmaceutiques et Biologiques (ISPB), Université Lyon 1, Lyon 69373, France; ^2^ INSERM UMR-S1052, CNRS UMR 5286, Université Lyon 1, Centre de Recherche en Cancérologie de Lyon (CRCL), LabEx DEVweCAN, Centre Léon Bérard, Lyon 69373, France; ^3^ Equipe labellisée Ligue 2014, BMSSI UMR 5086 CNRS-Université Lyon 1, IBCP, Lyon 69373, France; ^4^ EA 3738, Laboratoire de Ciblage Thérapeutique en Cancérologie, Centre Hospitalier Lyon-Sud, Pierre Bénite, France; ^5^ Hospices Civils of Lyon, Laboratoire de Biochimie de Lyon Sud (CBS), Lyon, France; ^6^ Univ. Grenoble Alpes/CNRS, DPM UMR 5063, F-38041 Grenoble, France

**Keywords:** ABCG2, MDR phenotype, Inhibitors, Chemosensitization

## Abstract

ABCG2 is responsible for the multidrug resistance (MDR) phenotype, and strongly modulates cancer outcomes. Its high expression at a number of physiological barriers, including blood-brain and intestinal barriers, impacts on drug pharmacokinetics parameters. We characterized MBL-II-141, a specific and potent ABCG2 inhibitor. Combination of 10 mg/kg MBL-II-141 with the anticancer agent CPT-11 completely blocked the growth of 90% freshly implanted ABCG2-positive tumors. Moreover, the same combination slowed the growth of already established tumors. As required for preclinical development, we defined the main pharmacokinetics parameters of MBL-II-141 and its influence on the kinetics of CPT-11 and its active metabolite SN-38 in mice. MBL-II-141 distribution into the brain occurred at a low, but detectable, level. Interestingly, preliminary data suggested that MBL-II-141 is well tolerated (at 50 mg/kg) and absorbed upon force-feeding. MBL-II-141 induced a potent sensitization of ABCG2-positive xenografts to CPT-11 through *in vivo* ABCG2 inhibition. MBL-II-141 strongly increased CPT-11 levels in the brain, and therefore would be a valuable agent to improve drug distribution into the brain to efficiently treat aggressive gliomas. Safety and other pharmacological data strongly support the reglementary preclinical development of MBL-II-141.

## INTRODUCTION

ATP-binding cassette (ABC) proteins secrete various endogenous and exogenous molecules to protect cells from xenobiotic- and toxin-induced alterations under physiological and pathological conditions. Three major ABC transporters, P-glycoprotein (P-gp/MDR1/ABCB1), multidrug resistance protein 1 (MRP1/ABCC1), and breast cancer resistance protein (BCRP/MXR/ABCG2), are overexpressed and widely contribute to the multidrug resistance (MDR) phenotype. The latter is also associated with a lower survival of cancer patients [1–4]. ABCG2 expression provides an early prediction of the clinical outcome of cancers, which can be used to improve the clinical management of gastric cancer patients [5]. Interestingly, expression levels of ABCG2 have been described as an important determinant of the “side-population” (SP) phenotype, characteristic of stem cells (as a Hoechst 33342-negatively stained population compared to the majority of cells) [6]. ABCG2-positive cells from hepatocellular carcinoma (HCC), displayed enhancements in tumorigenicity, proliferation capacity, doxorubicin resistance, cell migration, and invasion potential, whereas ABCG2 down-expression by siRNA significantly blocked these malignant properties [7]. Consequently, anticancer therapy could eliminate the majority of tumor cells, generate clonal selection of aggressive tumor cells overexpressing ABCG2 and cause their relapse. In conclusion, this suggests that the ABCG2 protein is an appealing therapeutic target, and inhibiting its mediated secretion or decreasing its expression level in cancer cells constitute potential strategies to overcome chemoresistance.

ABCG2 is responsible for the secretion of a large range of molecules, including anticancer agents such as mitoxantrone [8], topotecan, irinotecan and its SN-38 active metabolite [9, 10]. *In vitro* experiments showed that, by decreasing the intracellular retention of anticancer agents, ABCG2 drastically reduces drug cytotoxicity. In addition, since it is strongly expressed in polarized cells within physiological barriers, including the blood-brain barrier (BBB) [11], hepatocytes [12] and enterocytes [13], it also limits drug distribution within the body. ABCG2 is also expressed in the breast [14] and the kidney [12]. It is well recognized to influence the pharmacokinetics parameters of substrates such as topotecan [15], camptothecin [16], methotrexate (in combination with ABCC2 and ABCC3) [17], tandutinib [18], and erlotinib [19]. In consequence, inhibition of ABCG2 may strongly improve distribution of its substrates into the brain [20] and thus modulate brain tumor treatment. Recently, Kawamura *et al*. showed the role of ABCG2 and ABCB1 on CPT-11 (irinotecan) distribution inside mouse brain [21] which could be harnessed to improve efficiency of aggressive gliomas treatment. Strongly expressed at the physiological barriers, ABCG2 could also decrease plasma levels of anticancer drugs that are rapidly metabolized and excreted without reaching pharmacological concentration levels.

Whilst initial clinical assays were often discordant and not fully convincing, targeting ABC proteins with modulators remains a possible approach to improve chemotherapy efficiency. Chemosensitization strategies targeting ABCB1 have been so far unsuccessful due to low numbers of candidate inhibitors and unsuitable trials [22]. Inhibitors still need to be improved by increasing substrate specificity and lowering toxicity. Moreover, they should not show any adverse effect on drug pharmacokinetics that could lead to toxic plasma levels of anticancer drugs. The neurotoxic fumitremorgin C (FTC) was the first selective ABCG2 inhibitor identified [23]. Later, synthetic analogs of FTC were studied, including Ko143 which was devoid of toxicity and increased the oral availability of topotecan, giving plasma levels 4 to 6-fold higher in ABCB1(−/−) mice [24]. *In vitro*, other ABCG2-specific modulators were developed from the structure of the ABCB1 inhibitor tariquidar [25], although their *in vivo* efficiency over ABCG2-mediated chemoresistance has not been yet reported. Very recently, telatinib (15 mg/kg) in combination with doxorubicin (1.8 mg/kg) was shown to significantly decrease the growth rate and tumor size of ABCG2-overexpressing tumors in a xenografted nude mouse model [26]. In order to specifically study the impact of ABCG2 modulation on substrate pharmacokinetics and reversion of MDR phenotype, we designed an ABCG2-selective inhibitor (MBL-I-87) using the structure of the ABCB1 inhibitor elacridar as a template. It proved to be an efficient inhibitor of ABCG2 excretion [27] which improved *in vivo* CPT-11 anticancer activity on the growth of ABCG2-positive xenografted tumors [28, 29]. We also developed an inhibition model of tumor growth [30] predicting tumor growth dynamics, to be used as a template to design studies for producing more efficient ABCG2 inhibitors. Indeed, our team recently screened an original chemical library to identify more powerful inhibitors, some being devoid of toxicity. Using a chromone core instead of the acridone moiety, we identified a highly promising candidate for *in vivo* studies: MBL-II-141 or 5-(4-bromobenzyloxy)-2-(2-(5-methoxyindolyl) ethyl-1-carbonyl)-4H-chromen-4-one, also called chromone 6g [31]. MBL-II-141 displayed a high-affinity inhibition activity (IC_50_ of 0.11 μM) and a very low cytotoxicity (IG_50_ > 100 μM). It was shown to inhibit ABCG2 in various cell lines, either. Transfected with expression vector of ABCG2 or selected with mitoxantrone [32] and was found to be the most powerful modulator yet described. Similarly to other ABCG2-selective inhibitors such as acridones [27] and methoxy trans-stilbenes [33], MBL-II-141 inhibition is non-competitive towards mitoxantrone [31]. It is able to strongly inhibit the efflux of all tested substrates including mitoxantrone and SN-38 [31], pheophorbide A and BODIPY-prazosin [32]. Its remarkable high therapeutic index (> 900) completely justified its *in vivo* evaluation aimed at chemosensitizing ABCG2-positive tumors. In this study, we established a protocol of pharmacological treatment (*i.e*. dose and route of administration) based on the prediction model developed with MBL-I-87 [29] to evaluate the efficacy of MBL-II-141 against tumor growth. Furthermore, we looked at its tolerance in mice and we found that its pharmacokinetic parameters met the preliminary requirements for further clinical drug development.

## RESULTS

### MBL-II-141 *in vivo* sensitizes ABCG2-positive xenografts to CPT-11 treatment

*In vitro* studies previously demonstrated the valuable inhibitory effect of MBL-II-141 on ABCG2 drug-efflux activity. This compound was found to be a powerful and highly-specific inhibitor of ABCG2, blocking mitoxantrone efflux with an IC_50_ of 0.11 μM, without any effect on ABCC1-mediated transport using either mitoxantrone or calcein as substrates [31]. Furthermore, little effect on viability was observed *in vitro*, with less than 25% cell death of HEK293 cells at 100 μM; therefore, MBL-II-141 displayed a low cytotoxicity and appeared to be a promising molecule for *in vivo* assays of ABCG2 inhibition.

**Figure 1:**
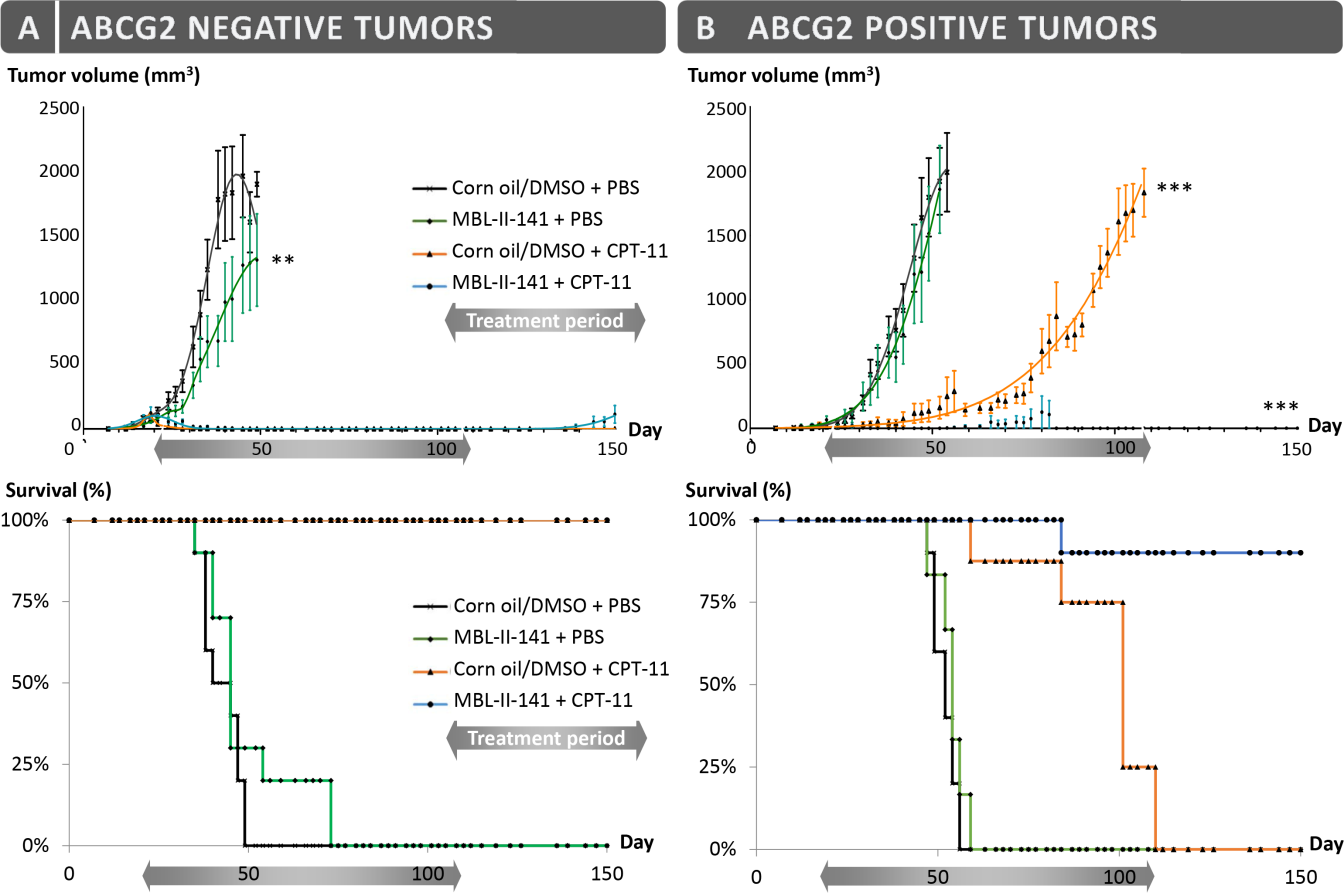
Effects of CPT-11 treatment in combination with MBL-II-141 on tumor growth and mice survival **(A)** HEK-pcDNA3 and **(B)** HEK-ABCG2 xenografts were implanted sub-cutaneously into mice. After onset of the tumors, mice were treated three times a week by: corn oil/0.5% DMSO + PBS (black crosses), MBL-II-141 at 10 mg/kg + PBS (green squares), corn oil/5% DMSO + CPT-11 at 20 mg/kg (orange triangles) and MBL-II-141 at 10 mg/kg + CPT-11 at 20 mg/kg (blue circles). The treatments started at day 21 and lasted until day 108. **P < 0.001

Firstly, we evaluated MBL-II-141 efficacy to chemosensitize ABCG2-positive, freshly established, tumors to CPT-11 treatment. Following the onset of tumors growing from HEK293-ABCG2 cells or control HEK293-pcDNA3.1 cells (implanted into the flank of SCID mice), the animals were treated by CPT-11, either alone or in combination with MBL-II-141. All xenografts grew rapidly in the presence of the vehicles (corn oil/5% DMSO and PBS) (Figure 1), while MBL-II-141 displayed a slight inhibitory effect on the growth of ABCG2-negative tumors (Figure 1A and Supplementary table 1). This effect was not observed for ABCG2-positive tumors (Figure 1B), and was not related to an enhanced cytotoxicity of MBL-II-141 on HEK293-pcDNA3.1 control cells. On the contrary, it was found to be slightly more cytotoxic *in vitro* on HEK293-ABCG2 cells than on HEK293-pcDNA3.1 cells (leading to 60% and 75% cell survival, respectively, at 100 μM) [31].

The CPT-11 treatment completely blocked the growth of ABCG2-negative cells, whereas it only delayed, by about two months, the growth of ABCG2-positive tumors. Interestingly, co-treatment of CPT-11 with MBL-II-141 was able to completely block the growth of 90% ABCG2-positive tumors. In terms of animal survival (Figure 1A), CPT-11 alone was sufficient to ensure the survival of 100% of mice bearing ABCG2-negative tumors. It also increased the survival of mice bearing ABCG2-positive tumors, although these mice had to be sacrificed at day 110 (Figure 1B) as the tumor size reached the maximal size authorized by the ethical code. The combination of MBL-II-141 and CPT-11 remarkably increased the survival rate of mice, up to 90%, even after 40 days following the end of the treatment (Figure 1B).

**Figure 2:**
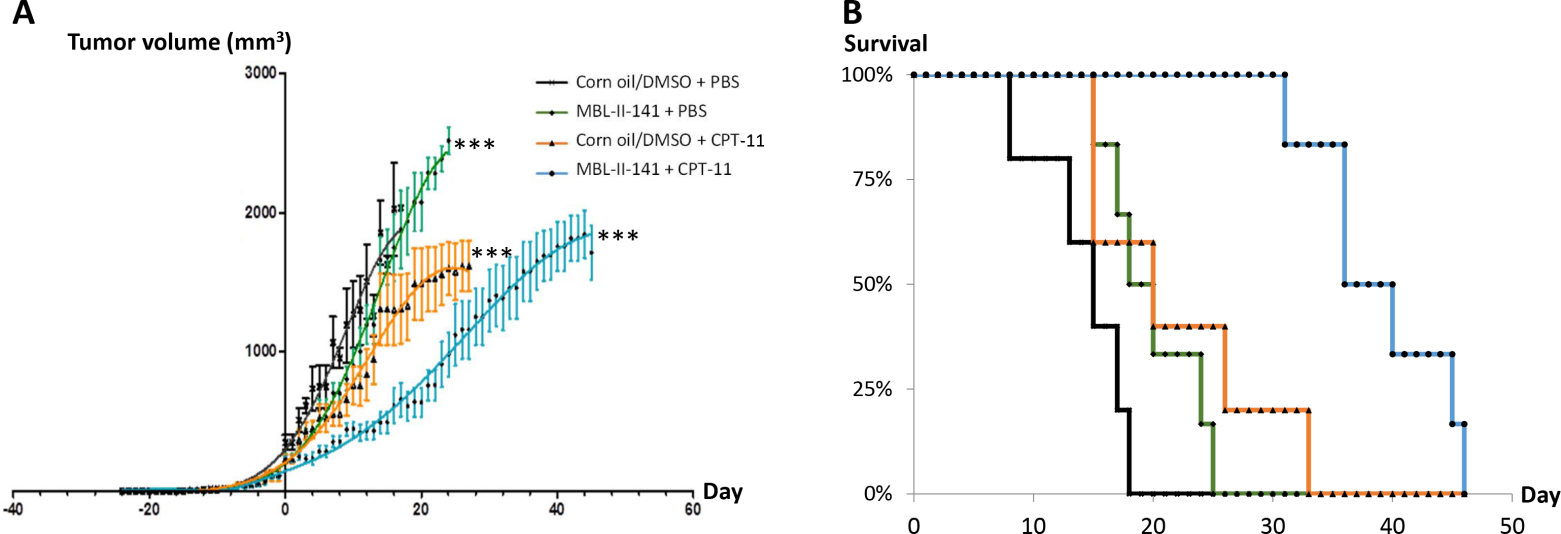
Effects of CPT-11 treatment in combination with MBL-II-141 on ABCG2-positive tumors of 8 mm diameter **(A)** Tumor growth curves and **(B)** Mice survival. HEK293 ABCG2 xenografts were implanted sub-cutaneously into mice. When tumors reached 8 mm in diameter, mice were treated three times a week by: corn oil/0.5% DMSO + PBS (black crosses), MBL-II-141 at 10 mg/kg + PBS (green squares), corn oil/5% DMSO + CPT-11 at 20 mg/kg (orange triangles) and MBL-II-141 at 10 mg/kg + CPT-11 at 20 mg/kg (blue circles). Treatment started at day 0. ***P < 0.001

Since the combination efficiently prevented the growth of early ABCG2-positive tumors, we then tested the effects of the same combination on established tumors (Figure 2). We delayed the pharmacological treatment until tumors reached 8 mm in diameter (mean volume of 238 mm^3^); this represented well-established tumors, fully developed and undergoing exponential growth. Treatments with MBL-II-141 alone had a negligible effect on tumor growth (Figure 2A), with a slope of 68 ± 3 mm^3^/day *versus* 61 ± 3 mm^3^/day for tumors treated with solvents. CPT-11 alone was able to delay the tumor growth, with a slope of 50 ± 2 mm^3^/day, but did not markedly improve mice survival (Figure 2B). Only the drug combination was able to greatly lower tumor growth, with a slope of 41 ± 1 mm^3^/day, and the tumors reached their maximal size about two weeks later than those treated with CPT-11 alone (Figure 2A). Consequently, the combination markedly increased mice survival (Figure 2B and Supplementary table 2).

### MBL-II-141 modifies CPT-11 and SN-38 pharmacokinetics

**Figure 3:**
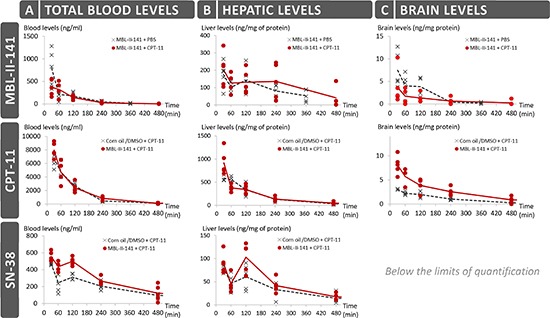
Pharmacokinetics studies of MBL-II-141 levels in total blood **(A)** liver **(B)** and brain **(C)** MBL-II-141 alone (at 10 mg/kg) or in combination with 20 mg/kg of CPT-11 was intraperitoneally administered to SCID mice. Blood, brain and liver were taken for LC-MS/MS analysis after 30, 60, 120, 240 or 360 min for treatments with MBL-II-141 alone, and after 30, 60, 120, 240 or 480 min for combination treatments (*N* = 5 mice/group).

From the perspective of putative pharmaceutical development, we fully analyzed the kinetics parameters of MBL-II-141 in SCID mice (Figure 3 and Supplementary table 3). The highest level of MBL-II-141 in total blood was observed at 0.5 h (Figure 3A). The levels then rapidly decreased to below the detection limit at 6 h. The elimination profile was similar in either the presence or absence of CPT-11 after 1 h. MBL-II-141 distribution into the brain was at a low, but still detectable, level (Figure 3C). CPT-11 appeared to decrease the MBL-II-141 distribution into the brain until 4 h, then MBL-II-141 levels became similar in either absence or presence of CPT-11.

While the CPT-11 injected dose was 2-fold higher than MBL-II-141 (20 *versus* 10 mg/kg), the CPT-11 blood levels were relatively much higher (Figure 3A), and the levels were globally 10-fold higher than those of MBL-II-141 at the same time points. 90% of CPT-11 was lost from the blood at 4 h. CPT-11 levels also rapidly decreased in the liver (Figure 3B). A very weak distribution was observed into the brain, with a value 200-fold lower than in the liver. However, the CPT-11 brain levels were significantly and durably increased in the presence of MBL-II-141 (Figure 3C), by approximately 2-fold (Supplementary table 4), whereas no impact was observed on the liver levels.

In parallel, we also quantified the levels of SN-38 in blood and tissues, which acts as both the CPT-11 active metabolite and an ABCG2 substrate. It was detected in blood until 6 h (Figure 3A), and the median level greatly decreased after 2 h. In the presence of MBL-II-141, SN-38 kinetics displayed a similar profile (with an increase of blood level at 1 h) but, interestingly, MBL-II-141 increased the SN-38 blood levels. This effect disappeared after 8 h, when levels became almost undetectable. In the liver, SN-38 followed a similar profile as in the blood, but the increase observed in the presence of MBL-II-141 was barely significant (Figure 3B and Supplementary Table 3), while SN-38 levels in the brain were below the limits of quantification (Figure 3C).

**Table 1:** Predictions of MBL-II-141 behavior by the ACD Percepta software Predictions were obtained according to data available for structurally related molecules. LD50: lethal dose 50; RBA: relative binding affinity (Log RBA) to a receptor, compared to the reference ligand estradiol; HSA: human serum albumin; PPB: percentage of a compound bound to human plasma proteins; V_D_: volume of distribution; BB: brain/blood distribution ratio; PS: rate of passive diffusion/permeability (logPS).

**Physicochemistry**		**Oral bioavailability**	
Molecular mass	547.40 g/mol	Bioavailability	9%
Index of refraction	1.68	Active transport	Not transported by PepT1 et ASBT
Density	1.48	**Passive absorption**	
logP	4.87		100% (transcellular)
**Acute toxicity**		**ABCB1 specificity**	
**Mouse LD50**		Inhibitor	Weak probability: 0.15 (Ki < 1μM) (RI = 0.18)
Intraperitoneal	290 mg/kg	Substrate	Weak probability: 0.03 (RI = 0.26)
Oral	1200 mg/kg	**Distribution**	
Intravenous	77 mg/kg	Protein Binding	Acid compound
Sub-cutaneous	320 mg/kg		Bound fraction to HSA; %PPB 99.68% (RI = 0.31)
**Rat LD50**			Unbound in plasma = 0.0032
Intraperitoneal	570 mg/kg	V_D_	3.5 L/kg
Oral	210 mg/kg	**Blood-Brain Barrier (BBB)**	
**Genotoxicity**		BBB	Low brain penetration
Ames test positivity	Weak probability: 0, 17	log BB	−0.41
**Irritation by local exposure**		log PS	−1.3
Eye irritation	Probability: 0.21		
Skin irritation	Probability: 0.25		
**Endocrine system: estrogen receptor (ER)**			
Binding to ER α	Weak probability (−3 < log RBA < 0)		

### MBL-II-141 is accumulated at low levels and is orally absorbed

**Figure 4:**
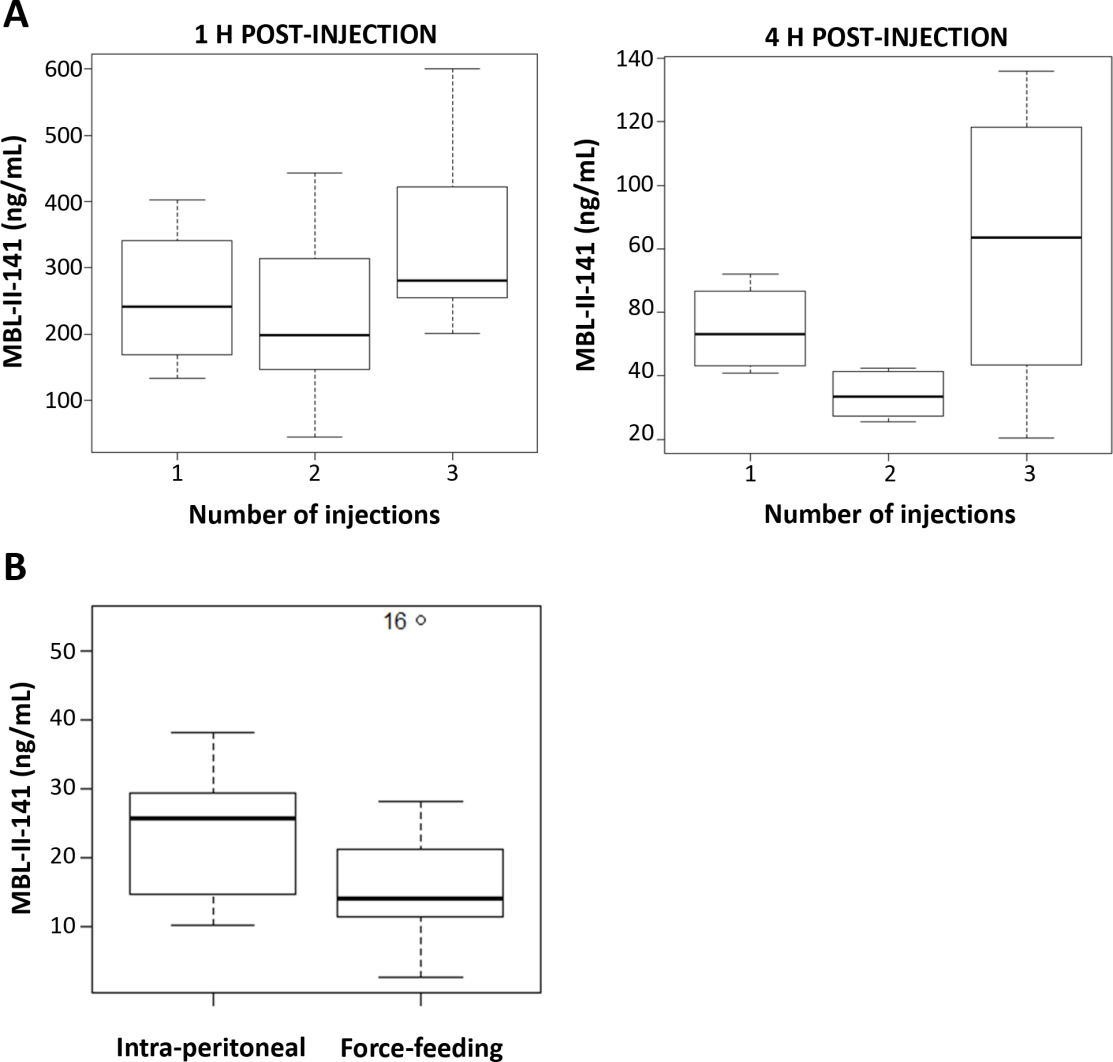
(A) Impact of multiple-injection treatments on MBL-II-141 plasma levels at 1 h or 4 h after the last injection MBL-II-141 was administered at 10 mg/kg to SCID mice by IP injection one, two or three times separated by 4 h intervals. Blood was withdrawn for HPLC-MS/MS analysis at 1 h or 4 h after the last injection (*N* = 6 mice/group). **(B)** Comparison of the route administration. MBL-II-141 at 10 mg/kg was administered by either IP injection or force-feeding. The results correspond to plasma levels observed 4 h after the administration (*N* = 7 mice/group). One value from the force-feeding treatment group was considered as an outlier by the R software, and so not considered in the analysis.

As previously observed, MBL-II-141 was rapidly eliminated from blood after a single injection. To study a possible accumulation effect, we quantified blood levels of MBL-II-141 at 1 or 4 h after the last injection in a multiple-administration protocol. MBL-II-141 blood levels were similar after one or two injection(s) (Figure 4A), while the third injection led to a slight increase of blood level, regardless of the time of blood collection (Figure 4A). In addition, we evaluated whether this molecule was orally absorbed upon force-feeding. The blood level was approximately 2-fold lower at 4 h under force-feeding in comparison to an intraperitoneal (IP) route (Figure 4C). Nevertheless, the observed difference was not significant due to the high variability of blood levels.

### MBL-II-141 is predicted to be a highly tolerated molecule

In view of the requirements of this new drug for pharmaceutical development, we investigated *in silico* safety data by using the Percepta software. By comparing *in vitro* experimental data from structurally-related molecules, it was possible to predict its oral absorption, tissue distribution, genotoxicity and cancerogenicity (Table 1). Percepta predictions indicated only a weak acute toxicity of MBL-II-141. In mice, the predicted LD50 was 290, 1200, 77 or 320 mg/kg, respectively, by IP, oral, intravenous or sub-cutaneous route. Similar data were predicted for rats, which are frequently used in preclinical studies (Table 1). In our pilot study, no acute toxicity was experimentally observed at 10 mg/kg per mouse. Furthermore, reiterated administrations confirmed MBL-II-141 safety without any associated animal death or loss of weight. Upon IP administration at 10, 30 or 50 mg/kg MBL-II-141 in combination with CPT-11 at 20 mg/kg, mice did not show any modification in behavior (physical activity, appearance). Complete blood count proportion was unchanged regardless of the administered dose of MBL-II-141 (data not shown), and macroscopic analysis of various organs (brain, heart, lungs, kidneys, liver and spleen) showed no apparent lesions. These results were confirmed with the Percepta software where the Ames test was negative, with and without metabolism systems. In the absence of structurally-related molecules in the Percepta software database, a low index of confidence was not achieved for prediction validation of complementary genotoxicity tests (micronucleus *in vitro* and *in vivo*). Only a weak probability was predicted for MBL-II-141 on its skin irritating nature and putative effect on the endocrine system via interaction with estrogen receptor alpha (ER). To complete the predicted safety of MBL-II-141, we observed that its structure would not permit transport either by PepT1 and ASBT, or by ABCB1. Moreover, it must bind with high affinity to human serum albumin (HSA) in blood, suggesting a low unbound fraction in plasma that represents the active fraction on the target. All these data were in full agreement with our preliminary data indicating that MBL-II-141 was orally absorbed (Figure 4) and not transported (35).

**Table 2:** Comparison of ABCG2 inhibitors described in the literature for *in vivo* experiments IC_50_: Inhibitory concentration of 50% of the ABCG2 activity; IG_50_: Inhibitory growth of 50% of the cell proliferation; I.V.: Intravenous; I.P.: Intraperitoneal; P.O.: Per Os.

Name	*In vitro*				*In vivo* Toxicity		*In vivo* Impact on	
	Inhibition (IC_50_)		ABCG2 Specificity	Cytoxicity (IG_50_)	Acute	Chronic	Tumor growth	Pharmacokinetics
GF120918 [13, 21, 35, 45–48]	0.31 μM	Mitoxantrone	No	1–40 mM	> 5 mg/kg I.V.	> 400 mg oral (human)	Yes	Doxorubicin : No
					> 10 mg/kg I.P.			Topotecan: Yes
					> 50 mg/kg oral			CPT-11: Yes
Ko143 [6, 24, 49]	0.22 μM	Hoechst 3342	Yes	18–34 μM	> 50 mg/kg oral			Topotecan: Yes
	0.35 μM	Pheophorbide A			> 10 mg/kg I.P.			
Telatinib [26, 50, 51]		[3H]-Mitoxantrone [3H]-E217BG	No	> 10 μM	>15 mg/kg oral	>1 500 mg (human)	Yes	
Triclabendazole [7]	≈ 10 μM	Mitoxantrone			> 100 mg/kg I.P.			Nitrofurantoin: Yes
Sorafenib [25]			No	2–6 μM	> 25 mg/kg oral	> 25 mg/kg P.O.	Yes	
YHO-13177 [40]	≈0.3 μM	Hoechst 3342	Yes	> 10 μM	> 200 mg/kg oral		Yes	
					> 30 mg/kg I.V.			
MBL-II-141	0.11 μM	Mitoxantrone	Yes	> 100 μM	> 10 mg/kg oral	> 10 mg/kg I.P.	Yes	CPT-11: Yes
					> 50 mg/kg I.P.	> 50 mg/kg I.P.		

## DISCUSSION

Chemosensitization targeting ABCG2 has been widely explored using inhibitors, and other strategies such as the use of shRNA [34]. Its combination with the anticancer agent 5-FluoroUracil reduced the size of tumors by 24% after a unique direct intratumoral injection. However, safety requirements of such a silencing system in clinical applications are complicated and under development. Thus, research of chemical molecules is still a valuable strategy. Our present study concerns the inhibitory effect of MBL-II-141 on ABCG2-mediated drug resistance to CPT-11. As expected, CPT-11 alone blocked the growth of ABCG2-negative xenografts, but only delayed the growth of ABCG2-positive xenografts. In contrast, its combination with MBL-II-141 is shown not only to completely block the growth of freshly implanted ABCG2-positive tumors, but also to strongly slow down the growth of already established tumors. ABCG2 inhibition by MBL-II-141 in ABCG2-positive xenografts therefore plays a significant and synergistic role with CPT-11 anticancer activity. With an IC_50_ of 0.11 μM, MBL-II-141 is known as one of the most powerful inhibitors ever described for ABCG2 (Table 2) [31, 32]. Its elevated IG_50_ (50% of cell growth inhibition), > 100 μM, leads to a high *in vitro* therapeutic ratio [31], and is consistent with low toxicity for mice in a repeated-administration protocol (with 10 mg/kg MBL-II-141 in combination with 20 mg/kg CPT-11 for more than 100 days). Several ABCG2 inhibitors have been tested *in vivo* (Table 2): some of them, such as GF120918 [35], Telatinib [26], Sorafenib [36] and YHO-13177 [37], have been described to delay the growth of small tumors in combination with doxorubicin or CPT-11. However, MBL-II-141 is clearly shown here to be the most effective, by completely inhibiting the growth of 90% tumors. In addition, its *in vivo* efficiency was validated at a relatively low dose (10 mg/kg), which also suggests a high inhibition activity in animal models. Moreover, MBL-II-141 is the first ABCG2 inhibitor ever described to significantly delay the growth of well-established tumors. Together with YHO-13177 [37] and Ko143 [24], MBL-II-141 is one of the very few ABCG2-specific inhibitors assayed in animal models. It is indeed selective for ABCG2, and not known to interact with other regulatory functions. Compared to the above inhibitors, MBL-II-141 still remains the most powerful as suggested by its inhibition activity (Table 2) and the complete disappearance of 90% tumors observed in the present study.

In light of ABCG2-positive tumor sensitization and as required by preclinical development, we decided to define the pharmacokinetics parameters of MBL-II-141 and its possible interactions on CPT11 and SN-38 pharmacokinetics. The ABCG2 transporter plays a key role in protecting the brain parenchyma by catalyzing the efflux of xenobiotics from capillary endothelial cells at the BBB. It largely prevents the entry of its anticancer substrates at the BBB, thereby limiting their efficacy [20, 38]. In addition, Kawamura *et al*. (2013) recently demonstrated that ABCG2 strongly decreased CPT-11 distribution into the brain [21] by using labeled [^11^C]-CPT-11 to specifically track CPT-11, even in the presence of its SN-38 active metabolite. In complete agreement with these data, and despite high blood levels, we also found that CPT-11 displayed an apparently low distribution into the brain. However, similarly to GF120918 (5 mg/kg) [21], MBL-II-141 significantly increased CPT-11 levels into the brain. The SN-38 blood levels were strongly increased by MBL-II-141, but remained under the limit of detection into the brain. In comparison with Lin's work with normal FVB mice, intravenous injection of CPT-11 led to lower plasma levels than those observed here for our immunodeficient SCID mice. In contrast, the brain levels were higher in these normal strain mice, and SN-38 was consequently detectable [16]. In ICR mice, Yamazaki *et al*. described SN-38 plasma levels 27-fold lower than CPT-11, 1 h after the injection, [37] which is consistent with our difference of 19-fold between CPT-11 and SN-38 levels. Taken altogether, the results show a lower brain distribution in our mouse strain; nevertheless, CPT-11 distribution is increased and can be locally metabolized to SN-38 [39] by brain carboxylesterases [40]. The results suggested that MBL-II-141 positively influences SN-38 distribution into the brain and tumors, increasing its antitumor effect. Nevertheless, from the perspective of clinical use, the distribution of SN-38 has to be completed by evaluating the possible SN-38 accumulation in blood after reiterated administration of CPT-11 in combination with MBL-II-141. Our preliminary data did not show any significant change in SN-38 blood level after a 2 h treatment with CPT-11 and MBL-II-141 whether the animals were submitted to an acute or a chronic treatment (Supplementary figure 2). Furthermore, no side effects (loss of weight, modification of blood count) were observed during the several months of combination treatments.

Consequently, MBL-II-141 would be useful to increase the distribution of CPT-11 into the brain and, potentially, to locally treat gliomas since CPT-11 has proved its antitumor activity against MDR glioblastoma cells [41]. This potential clinical development is also well supported by the fact that ABCG2 function at the BBE is relatively higher in human than in mouse. CPT-11 distribution may clearly be improved in the presence of MBL-II-141 in aggressive gliomas treatments. This is further supported by recent data showing that irinotecan in combination with temozolomide and bevacizumab produced promising results [41].

From the perspective of a putative pharmaceutical development, our preliminary data indicate that MBL-II-141 is absorbed after force-feeding to a lesser extent than through the IP route. MBL-II-141 bioavailability was predicted to be around 9% (Table 1), consistent with our data after force-feeding. As likely due to its high hydrophobicity, MBL-II-141 was predicted to strongly bind to human serum albumin (HSA) in blood, suggesting a low unbound active fraction in plasma. Even though reglementary guidelines generally do not require such knowledge in cancer use (for treating aggressive tumors with a low survival rate), the Percepta software predicted that MBL-II-141 is not genotoxic and not carcinogenic. In agreement with data from our pilot experiments, MBL-II-141 displays a low toxicity level during both acute and repeated-administration protocols. Nevertheless, several injections in a short period of time increased CPT-11 blood level. Indeed, two injections separated by 4 h did not change the blood level, while the third injection led to a slight increase. This may be due to an accumulative effect in the peritoneal cavity, due to the high hydrophobicity, and possible precipitation, of MBL-II-141. This last hypothesis was supported by the fact that, at 50 mg/kg for 3 times a week for 1 month, a slight deposit in the peritoneal cavity was observed (data not shown). Furthermore, no sign of toxicity (such as decrease of weight and modification of behavior) has ever been observed.

To conclude, by inhibiting the *in vivo* transport activity of ABCG2, MBL-II-141 has proved its efficiency of chemosensitization in xenograft models, and is therefore of interest for improving drug distribution into the brain. All observations and prediction data suggest a high mouse tolerance to MBL-II-141, and indicate that it would indeed be suitable for clinical development towards resistant cancer therapy or treatment of gliomas, which are limited by physiological barriers where the ABCG2 transporter plays a central role.

## METHODS

### Products

MBL-II-141 was synthetized by Prof. A. Boumendjel (UMR 5063, Grenoble, France) as previously described [31]. Corn oil (#C8267-500ML) and DMSO (#D2650-100ML) were purchased from Sigma Aldrich (France). Matrigel (#734-1100) was obtained from VWR. DMEM (#E15-883), penicillin/streptomycin (#P11-010) and G418 (#P11-012) were supplied by GE Healthcare Life Sciences (France). FBS (#ES98PS) was obtained from Abcys. CPT-11 and heparin were kindly provided by Dr. C. Rioufol (Hospices Civils of Lyon, France).

### Cell lines

The human embryonic kidney (HEK) 293 cells were transfected as previously described [42]. HEK293 pcDNA3.1 (empty vector) and HEK ABCG2 (pcDNA3 vector coding for ABCG2) cell lines were cultured in DMEM (4.5 g/L glucose) supplemented with 10% FBS, 1% penicillin/streptomycin and 0.75 mg/mL G418. These cell lines are weakly tumorigenic [28].

### Animal experiments

All *in vivo* experiments were conducted by accredited researchers (Drs. L. Payen and M. Honorat) in accordance with the animal care guidelines of the European Union and French laws, and were validated by the local Animal Ethic Evaluation Committee (CECCAPP). The investigation was conducted in accordance with the ethical standards and according to the Declaration of Helsinki and to national and international guidelines and has been approved by the authors' institutional review board. Four week-old female mice (Charles River Laboratories) were maintained in a specific pathogen-free animal facility AniCan at the Centre Léon Bérard (agreement n° B 69 388 0202, Lyon, France). Mice were randomly distributed into groups.

The experimental formulation of MBL-II-141 was determined according to its solubility properties. MBL-II-141 [31] was dissolved in DMSO (maximal solubility of 250 mg/mL), and 20-fold diluted in sterile corn oil [43, 44].

At the end of experiments, total cardiac blood was collected either from animals intraperitoneally treated with 50 U.I./kg heparin (50 μL/mouse) for 1.5 h before withdrawal of blood samples, or using dried heparinized syringes to completely block clotting. Plasma was separated from blood cells by centrifugation (for 30 s at 2000 g). Blood and plasma were stored at −80 °C until drug quantification by LC-MS/MS.

#### MBL-II-141 tolerance assay

As recommended in the guidelines of The International Conference on Harmonisation of Technical Requirements for Registration of Pharmaceuticals for Human Use (ICH), the pilot tolerance study was conducted by using three doses of MBL-II-141 in combination with the anticancer agent CPT-11. SCID mice were randomly distributed into three groups (*n* = 10) according to the MBL-II-141 doses: 10 mg/kg (low concentration), 30 mg/kg (intermediate concentration) or 50 mg/kg (maximal solubility). They were intraperitoneally treated three times a week with MBL-II-141 and CPT-11 (20 mg/kg) for one month.

#### MBL-II-141 efficacy of chemosensitization on just-established tumors

4.10^6^ HEK-pcDNA3.1 or HEK-ABCG2 cells were sub-cutaneously injected into one flank of each SCID mouse, in solution with 50% matrigel. After the apparition of most tumors, mice were randomly distributed into 4 groups of 20 animals (10 ABCG2 negative and 10 ABCG2-positive tumors). In detail, group 1 received only vehicles (PBS and corn oil/5% DMSO); group 2 received only MBL-II-141 (10 mg/kg and PBS); group 3 received only CPT-11 (20 mg/kg and corn oil/5% DMSO) and group 4 received both molecules (MBL-II-141 at 10 mg/kg and CPT-11 at 20 mg/kg). Two independent injections were carried out per mouse.

Treatments were intraperitoneally administered three times a week until mice sacrifice. To evaluate the toxicity impacts of treatments, three times a week, mice were weighed and their behaviour was observed; tumor size was measured with a caliper in two perpendicular dimensions. Finally, mice were sacrificed when the tumor diameter reached 17 mm.

#### Efficacy of chemosensitization on well-established tumors

1.10^6^ HEK ABCG2 cells were sub-cutaneously injected into one flank of each SCID mouse, in solution with 30% matrigel. Once the tumor reached 8 mm in diameter (268 mm^3^ total volume), as previously described, mice were treated either by vehicles (PBS and corn oil/5% DMSO), MBL-II-141 (10 mg/kg and PBS), CPT-11 (20 mg/kg and corn oil/5% DMSO) or with the combination (MBL-II-141 at 10 mg/kg and CPT-11 at 20 mg/kg). Each group contained 10 mice. The same protocol of treatment and follow-up were applied.

#### MBL-II-141 pharmacokinetic study

SCID mice (*n* = 6 per group) were intraperitoneally treated by MBL-II-141 either alone (10 mg/kg) or in combination with CPT-11 (20 mg/kg). MBL-II-141 was administered for 0.5, 1, 2, 4 or 6 h. CPT-11 was administered either alone or in combination with MBL-II-141 for 0.5, 1, 2, 4, 8 and 24 h. In order to achieve possible cumulative effects, reiterated administrations (two or three) were carried out with a 4 h time interval between administrations. Blood samples were collected at either 1 h or 4 h after the last injection.

Total blood samples were collected using dried heparinized syringes, avoiding modification of samples. Blood and plasma were stored at −80°C. Brain and liver were collected and stored at −80°C for LC MS/MS quantification of MBL-II-141, CPT-11 and SN-38. Tissues were crushed using a dried cold system.

#### MBL-II-141 oral absorption study

The availability of MBL-II-141 was evaluated by force-feeding (*n* = 4), and compared to intraperitoneal injection (*n* = 4). CD1 mice were treated with 10 mg/kg of MBL-II-141. After 4 h of treatment, total blood was collected from animals treated with heparin. Samples were stored at −80°C until quantification.

### HPLC-MS/MS analysis

CPT-11, SN-38 and MBL-II-141 were quantified in whole blood and in tissues by HPLC MS/MS (ThermoElectron, San Jose, USA). One assay was used for CPT-11 and SN-38 using gradient of elution with water-formic acid 0.1% and acetonitrile-formic acid 0.1%. Another gradient of elution was used for MBL-II-141 using ammonium acetate buffer (pH 6.5, 50 mM), acetonitrile and propanol-2. Both assays were performed on a C18 column HypersilGold, (ThermoElectron), 3.0 μm, (100 mm × 2.1 mm i.d.). The mass spectrometry analysis was performed in positive ion mode with electrospray source. Labeled CPT-11, SN-38 and MBL-II-141 were used as internal standards. CPT-11 and ^13^C_6_-CPT-11 were quantitated in selected reaction monitoring (SRM) mode using the transition m/z 587→124 and 593→124, respectively. SN-38 and ^13^C_6_-SN-38 were quantitated in SRM mode using the transition m/z 393→349 and 399→355, respectively. MBL-II-141 and ^2^H_3_-MBL-II-141 were quantitated in SRM mode using the transition m/z 547→342 and 550→345, respectively. A volume of 50 μL of whole blood or tissue solution was introduced into a conic centrifuge and 50 μL of solution of the internal standard and 300 μL of cold acetonitrile were added. The sample was mixed and centrifuged for 10 min at 13000 g at 5°C. For MBL-II-141, 250 μL of the clear supernatant was removed and evaporated to dryness under a stream of nitrogen. The residues were resuspended in 100 μL of mobile phase and 10 μL were injected into the HPLC device. For CPT-11 and SN-38, 50 μL of clear supernatant were diluted (1/5) in mobile phase and 10 μL were injected into the chromatographic device. For MBL-II-141, calibration curves were from 5 to 1000 ng/mL for whole blood and tissues analysis. For CPT-11 and SN-38, calibration curves were from 50 to 5000 ng/mL for whole blood and liver and from 50 to 500 ng/mL for brain. When the concentration was over the upper standard concentration, a dilution of the sample was performed using blank whole blood or appropriate tissues. Peak area ratios of compound to the respective internal standard measured at each nominal concentration were used to construct weighted (1/concentration) least-square linear regression curves. For the three compounds, the within-run precision and the between-run precision of the assay was less than 10%. Assay accuracy was in the range of 91–108%. The lower limit of quantification of compounds in medium was set at the lowest standard concentration. Results in tissues were normalized to cellular protein content. Results in tissues were normalized to cellular protein content (C1600 Abott technology).

### Prediction by ACD Percepta software

Predictions of some ADMET (absorption, distribution, metabolism, and excretion - toxicity) properties were computed using the ACD Percepta software, version 14.0.0 running on an intel i7 CPU laptop computer under windows 7. The default set and the ACD database was used without any modifications. The software uses proprietary set of QSAR (quantitative structure-activity relationship) equations to predict ADMET properties. The software is able to propose a reliable index (RI) made on structurally-related compounds found by a Tanimoto indice. The ACD standard library was used as database.

### Statistical analysis

*Growth models*. Tumor growth curves were modeled with GraphPad© Software. Gaussian distribution models were obtained according to the equation Y = Amplitude*exp (−0.5*((X-Mean)/SD)^2). The amplitude was the height of the center of the distribution in Y units. Mean was the X value at the center of the distribution. SD was a measure of the width of the distribution, in the same units as X. Another model was applied for sum of two Gaussian distributions with Y = one + two (One = Amplitude 1*exp(−0.5*((X-Mean1)/SD1)^2) and Two = Amplitude 2*exp(−0.5*((X-Mean2)/SD2)^2)). Amplitudes were the heights of the center of the distribution in Y units. Means were the X values at the center of the two distributions. SDs were measures of the widths of the distributions, in the same units as X.

*Paired t-test*. The statistical test used was the paired t-test. It was applied for all data of growth tumor experiments and pharmacokinetics data.

## SUPPLEMENTARY TABLES AND FIGURES


