# Supplementary material for: MBL-II-141, a chromone derivative, enhances irinotecan (CPT-11) anticancer efficiency in ABCG2-positive xenografts
Source: Oncotarget. 2015 Jan 20;5(23):11957–70. doi: 10.18632/oncotarget.2566 (PMC4323000; doi:10.18632/oncotarget.2566)
Supplement: Supplementary file 1 [file oncotarget-05-11957-s001.pdf]

SUPPLEMENTARY TABLES AND FIGURES

Supplementary Table S1: Significance of the differences observed between the growth of tumors treated by either vehicles (corn oil/5% DMSO and PBS) or MBL-II-141 alone (at 10 mg/kg and PBS). Tumors were treated just after their onset. The growth curves of tumors were submitted to paired t-test, as described in the Methods section.

	HEK-pcDNA3.1	HEK-ABCG2
**P value (two tailed)**	**0.0006**	**0.015**
t	4.220	2.688
df	17	18
Number of pairs	18	19
**Mean of differences**	**342**	**77.51**
95% confidence interval	171.0 to 512.9	16.93 to 138.1
R squared	0.5116	0.2864
Correlation coefficient (r)	0.629	0.9499
P value (one tailed)	< 0.0001	< 0.0001

Supplementary Table S2: Significance of the differences observed between the growth of ABCG2-positive tumors. Tumors were treated when one reached a diameter of 8 mm. Growth curves of tumors were submitted to paired t-test, as described in the Methods section.

	**Solvents** *versus* **MBL-II-141**	**Solvents** *versus* **CPT-11**	**Solvents** *versus* **MBL-II-141 + CPT-11**	**MBL-II-141** *versus* **MBL-II-141 + CPT-11**	**CPT-11** *versus* **MBL-II-141 + CPT-11**
**P value (two tailed)**	**< 0.0001**	**< 0.0001**	**< 0.0001**	**< 0.0001**	**< 0.0001**
t, df	4.773	4.641	4.553	5.009	5.909
df	41	41	41	48	51
Number of pairs	42	42	42	49	52
**Mean of differences**	**94.84**	**170.7**	**318.6**	**401.4**	**245.8**
95% confidence interval	54.71 to 135.0	96.40 to 245.0	177.3 to 460.0	240.1 to 562.7	162.2 to 329.4
R squared	0.3572	0.3444	0.3358	0.3433	0.4064
Correlation coefficient (r)	0.9658	0.8997	0.9062	0.9248	0.8922
P value (one tailed)	< 0.0001	< 0.0001	< 0.0001	< 0.0001	< 0.0001

Supplementary Table S3: Significance of the differences observed in CPT-11, SN-38 or MBL-II-141 levels. Mice were treated by CPT-11 and MBL-II-141 either alone or in combination. The levels obtained in blood, brain and liver were compared for mice treated with each compound or with their combination. The levels quantified over time were submitted to paired t-test as described in the Methods section.

	Blood	Brain	Liver
**CPT-11** (CPT-11 *versus* CPT-11 + MBL-II-141)			
**P value (two tailed)**	**0.3616**	**0.0416**	**0.6878**
t, df	1.004	2.724	0.4260
df	5	5	5
Number of pairs	6	6	6
**Mean of differences**	**-220.0**	**-2.085**	**-28.86**
95% confidence interval	-783.7 to 343.6	-4.053 to -0.1168	-203.0 to 145.3
R squared	0.1677	0.5974	0.03503
Correlation coefficient (r)	0.9540	0.9330	0.7830
P value (one tailed)	< 0.0001	< 0.0001	0.0013
**SN-38** (CPT-11 *versus* CPT-11 + MBL-II-141)			
**P value (two tailed)**	**0.0299**		**0.1727**
t, df	3.300		1.658
df	4		4
Number of pairs	5		5
**Mean of differences**	**-109.6**		**-12.65**
95% confidence interval	-201.8 to -17.42		-33.83 to 8.533
R squared	0.7314		0.4072
Correlation coefficient (r)	0.8739		0.8318
P value (one tailed)	0.0005		0.0014
**MBL-II-141** (MBL-II-141 *versus* CPT-11 + MBL-II-141)			
**P value (two tailed)**	**0.1555**	**0.1059**	**0.1046**
t, df	1.671	1.970	1.980
df	5	5	5
Number of pairs	6	6	6
**Mean of differences**	**122.0**	**38.53**	**-39.78**
95% confidence interval	-65.65 to 309.6	-11.75 to 88.80	-91.44 to 11.88
R squared	0.3584	0.4371	0.4394
Correlation coefficient (r)	0.5701	0.5008	0.3638
P value (one tailed)	0.0427	0.0848	0.1679

Supplementary Table S4: Changes in CPT-11, SN-38 or MBL-II-141 levels as induced by the combination treatment. Liver or brain levels were related to plasma levels in each mouse. Fold-change values were expressed regarding ratios obtained for treatment with one molecule (i.e. MBL-II-141 or CPT-11) compared to those obtained for treatment with combined molecules.

Time (min)	Liver/Plasma ratio			Brain/Plasma ratio	
	CPT-11	SN-38	MBL-II-141	CPT-11	MBL-II-141
30	1.3	0.9	2.5	2.2	1.1
60	0.7	0.6	0.8	2.6	0.3
120	1.1	1.1	1.1	2.2	0.4
240	0.6	1.0	3.0	1.4	3.0
480	1.8	1.0	N.D.	4.3	N.D.

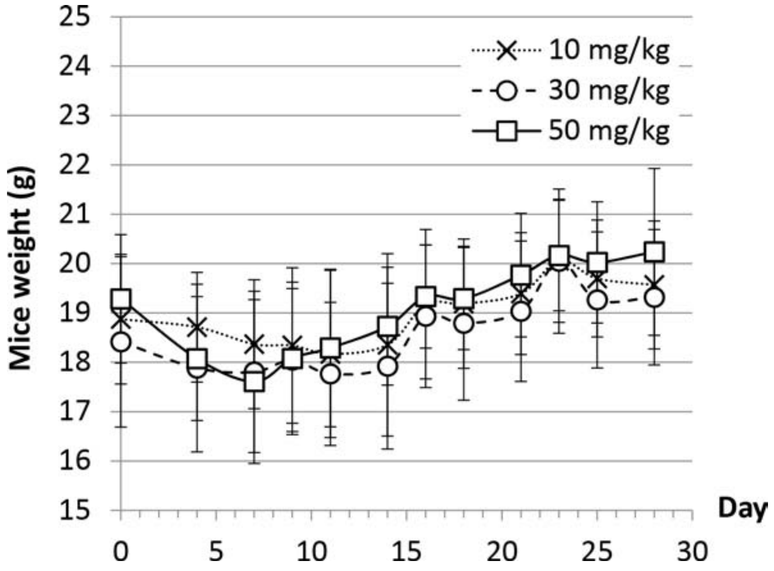

Supplementary Figure S1: Effects of MBL-II-141 on mice weight over a one-month treatment. MBL-II-141 was administered at 10, 30 or 50 mg/kg, 3 times a week, through intraperitoneal injections.

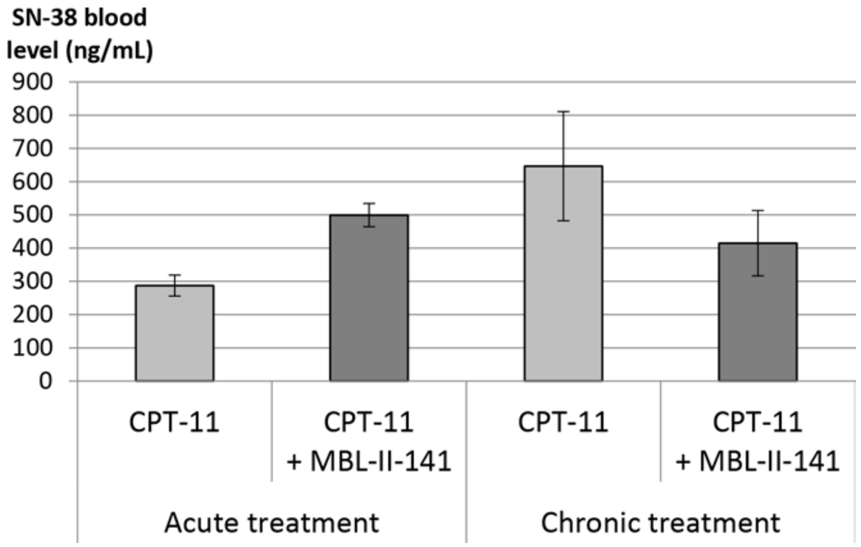

Supplementary Figure S2: Effects of MBL-II-141 chronic treatment on SN-38 blood levels. MBL-II-141 (10 mg/kg) in combination or not with CPT-11 (20 mg/kg) was administered to animals chronically treated (3 times a week for 40 days approximately, through intraperitoneal injections) or to acute treatment. SN-38 blood levels were quantified two hours after the last injection.
